# Retina regeneration in the chick embryo is not induced by spontaneous Mitf downregulation but requires FGF/FGFR/MEK/Erk dependent upregulation of Pax6

**Published:** 2007-01-24

**Authors:** Jason R. Spence, Mayur Madhavan, Juan-Carlos Aycinena, Katia Del Rio-Tsonis

**Affiliations:** Department of Zoology, Miami University, Oxford, Ohio

## Abstract

**Purpose:**

To elucidate the early cellular events that take place during induction of retina regeneration in the embryonic chick, focusing on the relationship between fibroblast growth factor (FGF) signaling and the regulation of Pax6 and Mitf.

**Methods:**

The retina of embryonic day 4 (E4) chicks was removed and a heparin coated bead soaked in fibroblast growth factor 2 (FGF2) was placed into the optic cup. The pharmacological inhibitor PD173074 was used to inhibit FGF receptors, PD98059 was used to inhibit MAP kinase-kinase/extracellular signal-regulated kinase (MEK/Erk) signaling. Retroviral constructs for paired box 6 (Pax6), MEK, and microphthalmia (Mitf) were also used in overexpression studies. Immunohistochemistry was used to examine pErk, Pax6, Mitf, and melanosomal matrix protein 115 (MMP115) immunoreactivity and bromodeoxyuridine (BrdU) incorporation at different time points after removing the retina.

**Results:**

The embryonic chick has the ability to regenerate a new retina by the process of transdifferentiation of the retinal pigment epithelium (RPE). We observed that during the induction of transdifferentiation, downregulation of Mitf was not sufficient to induce transdifferentiation at E4 and that FGF2 was required to drive Pax6 protein expression and cell proliferation, both of which are necessary for transdifferentiation. Furthermore, we show that FGF2 works through the FGFR/MEK/Erk signaling cascade to increase Pax6 expression and proliferation. Ectopic Mitf expression was able to inhibit transdifferentiation by acting downstream of FGFR/MEK/Erk signaling, likely by inhibiting the increase in Pax6 protein in the RPE.

**Conclusions:**

FGF2 stimulates Pax6 expression during induction of transdifferentiation of the RPE through FGFR/MEK/Erk signaling cascade. This Pax6 expression is accompanied by an increase in BrdU incorporation. In addition, we show that Mitf is spontaneously downregulated after removal of the retina even in the absence of FGF2. This Mitf downregulation is not accompanied by Pax6 upregulation, demonstrating that FGF2 stimulated Pax6 upregulation is required for transdifferentiation of the RPE. Furthermore, we show that ectopic Mitf expression is able to protect the RPE from FGF2 induced transdifferentiation by inhibiting Pax6 upregulation.

## Introduction

Regeneration of the retina occurs through the process of transdifferentiation in chick embryos and is limited to a small window of development between E3.5 and E4.5. Transdifferentiation is the process by which RPE cells lose their normal characteristics, becoming "stem cell-like." Retina removal is characterized by a thickening of the RPE, and upon addition of ectopic fibroblast growth factor 2 (FGF2), the RPE proliferates and gives rise to a neuroepithelium, which will subsequently differentiate into all of the different cell types of the retina [1-3].

Transdifferentiation of the RPE in the embryonic chick has been well characterized [1,4-6] and several studies using developing eyes from different species have demonstrated that transdifferentiation can be induced by FGF family members [7-9] and by constitutive activation of downstream effectors of the FGF pathway such as MAP kinase-kinase (MEK) [10].

Other stimulators of transdifferentiation analyzed during eye development include transcription factors such as Ceh-10 homeodomain-containing homolog (Chx10) and paired box 6 (Pax6), which are essential for retina development [11-17]. One way these factors may induce transdifferentiation is by downregulating microphthalmia (Mitf), a transcription factor required for proper RPE development. Mitf downregulation has been shown to be important in the transdifferentiation process. It has been demonstrated in quails and mice with Mitf mutations that the RPE will undergo a conversion to neural retina [8,18-20]. In addition, in mice lacking Chx10, ectopic Mitf expression was seen in the retina, suggesting one of the roles for Chx10 is to repress Mitf [17].

Pax6 is required for transdifferentiation of chick RPE cells into retina cells in vitro. During this in vitro transdifferentiation, Pax6 expression is upregulated while Mitf expression is downregulated. Similarly, overexpression of Mitf can inhibit the expression of Pax6 in cultured RPE [21]. These observations are suggestive of a close regulation between Pax6 and Mitf. Moreover, Pax6 and Mitf were shown to inhibit each other's transcriptional activity by direct protein-protein interactions in vitro [22]. These studies clearly point to a complex regulatory network between FGF signaling and Mitf, Chx10, and Pax6 transcription factors during RPE development.

Recently, it was shown that Pax6 is able to induce RPE transdifferentiation when overexpressed in chick eyes at embryonic stages of development as late as E14 [23]. Thus, even in the absence of ectopic FGF, and at stages that are normally not permissive for transdifferentiation, overexpression of Pax6 was able to stimulate transdifferentiation. In this context, Pax6 can be considered a "master regulator" of transdifferentiation.

However, it remains unclear why the RPE is capable of responding to FGF signals at E4 and not E5. It is the purpose of this work to closely examine early events of FGF-stimulated transdifferentiation in E4 RPE to lay the ground work for understanding why this process is so temporally restricted during development. Specifically, we show that during retina regeneration FGF acts through FGF receptors to stimulate Erk phosphorylation, and that this phosphorylation event is dependant on MEK activity, demonstrating that transdifferentiation occurs through the FGF-FGFR-MEK-Erk pathway. Furthermore, we show that one of the earliest events that takes place in RPE exposed to FGF2 is an upregulation of Pax6 protein as well as an increase in cell proliferation demonstrated by BrdU incorporation. We also show that Pax6 upregulation is not necessary for Mitf downregulation in the RPE during induction of transdifferentiation, but that Mitf overexpression is able to inhibit the early inductive events characteristic of transdifferentiation.

## Methods

### Chick embryos

White Leghorn fertilized eggs were purchased from the Ohio State University, Columbus, OH and incubated in a humidified incubator at 38 °C.

### Surgical procedures

A window was made in the egg shell using forceps, and microsurgical removal of the retina was carried out at E4 following guidelines previously described in references [1,4,5].

### Preparation of FGF2

Heparin-coated polyacrylamide beads (Sigma, St. Louis, MO) were washed 3 times in 1x PBS. FGF2 (R&D Systems, Minneapolis, MN) was resuspended in 1x PBS at a concentration of 1 μg/μl. Heparin beads were then incubated with FGF2 for at least 2 h before use.

### Rcas virus production and viral infection

Rcas-Mitf, Rcas-Pax6, constitutively active Rcas-MEK^DD^ and Rcas-GFP (control) were prepared using a procedure previously described in reference [1]. Rcas-Mitf was a kind gift from M. Mochii, Himeji Institute of Technology, Hyogo, Japan. Rcas-Pax6 was a kind gift from P. Bovolenta, Cajal Institute, Madrid, Spain. Rcas-MEK^DD^ was a kind gift from A. Eychene, Institut Curie, Orsay Cedex, France. Rcas-GFP was a generous gift from T. Belecky-Adams, IUPUI, Indianapolis, IN and R. Adler, Johns Hopkins University, Baltimore, MD. Retinectomies and Rcas infections were performed at E4 following procedure previously described in reference [1]. The embryos were placed back in the incubator for different lengths of time (3 or 7 days) at which time they were collected and processed for further experimentation.

### Tissue fixation and sectioning

Tissues used for immunohistochemistry were fixed in 4% formaldehyde, cryoprotected in 30% sucrose and embedded in O.C.T. freezing medium (Sakura Finetek, Torrance, CA). Eyes used for immunohistochemistry were sectioned at 10 μm.

### Antibodies

The anti-Pax6 and AMV3C2 antibodies were obtained from the Developmental Studies Hybridoma Bank developed under the auspices of the NICHD and maintained by The University of Iowa, Department of Biological Sciences, Iowa City, IA. Rat anti-BrdU and mouse anti-Mitf were purchased from Abcam (Cambridge, MA). Rabbit anti-pErk was purchased from Cell Signaling (Danvers, MA). The rabbit anti-Mitf was a kind gift from S. Saule, Centre Universitaire, France. The anti-MMP115 was a kind gift from M. Mochii, Himeji Institute of Technology, Hyogo, Japan. Secondary antibodies goat anti-rat Alexa 488, goat anti-rat Alexa 546, goat anti-rabbit Alexa 488, goat anti-rabbit Alexa 546, goat anti-mouse Alexa 488 were purchased from Molecular Probes (Eugene, OR).

### Antibody blocking experiments

The antibody to functionally block FGFR1 and FGFR2 was purchased from Abcam. To deliver the antibody, Affi-Gel Blue beads (Biorad, Hercules, CA) were washed several times in 1X PBS and then dehydrated through an ethanol series. Finally, an eppendorf tube containing beads suspended in 100% ethanol was placed on a heating block until the ethanol had evaporated completely. The antibodies were then added to the beads.

### Inhibition experiments

The FGFR inhibitor, PD173074 (Pfizer) and the MEK inhibitor, PD98059 (Calbiochem, San Diego, CA), were resuspended in DMSO at concentrations of 50 μm and 5 mM, respectively. The solutions were incubated in ethanol dehydrated Affi-Gel Blue beads as described above.

### Immunohistochemistry

A general immunohistochemical protocol was used as previously described [1]. Briefly, sections were washed, incubated overnight in primary antibody, washed, incubated for two hours in secondary antibody and mounted for fluorescent microscopy. All experiments were repeated in triplicate on at least three different eyes.

### Quantitation of Pax6, BrdU, and Pax6/BrdU positive cells in the RPE

Pax6 and BrdU immunohistochemistry was performed as described. Pax6 and BrdU images of the RPE close to the bead were taken using confocal microscopy. The total number of Pax6 and BrdU as well as Pax6/BrdU positive cells were counted in each section. The length of RPE from which the cells were counted was also measured. The number of cells counted in a section of RPE was divided by the section's length to determine the number of Pax6, BrdU, or Pax6/BrdU positive cells per micrometer of RPE. Sections from three different eyes were used. The Student-t-test was used to determine statistical significance.

## Results

### MEK and Pax6 stimulate transdifferentiation while Mitf inhibits transdifferentiation after retinectomy

**Figure 1:**
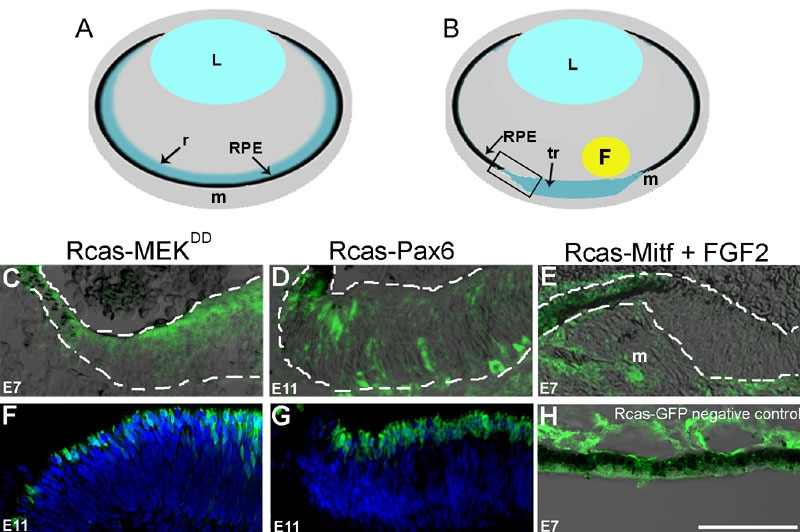
Stimulation and inhibition of transdifferentiation. **A**: Schematic of an intact eye at E4. **B**: Schematic of a transdifferentiating eye three days after retina removal. Boxed area demonstrates orientation of **C**-**H**. F represents FGF2 bead. All eyes underwent retinectomy at E4 (**C**-**H**). **C**: Infection of the RPE with Rcas-MEK^DD^ at E4 induced transdifferentiation by E7. AMV3C2 staining represents the presence of the Rcas virus in the RPE. **D**: Infection of the RPE with Rcas-Pax6 at E4 induced transdifferentiation by E11. AMV3C2 staining represents the presence of the Rcas virus in the RPE. **E**: Infection of the RPE with Rcas-Mitf at E4 is able to inhibit transdifferentiation stimulated by FGF at E7. AMV3C2 staining represents the presence of the Rcas virus in the RPE. Only areas of the RPE that were not infected with Rcas-Mitf were able to transdifferentiate. **F**: Infection of the RPE with Rcas-MEK^DD^ at E4 induced transdifferentiation by E11. Some of the transdifferentiated RPE started to differentiate and express the photoreceptor marker visinin (green). Tissue was counterstained with DAPI (blue). **G**: Infection of the RPE with Rcas-Pax6 at E4 induced transdifferentiation by E11. Some of the transdifferentiated RPE starts to differentiate and express the photoreceptor marker visinin (green). Tissue is counterstained with DAPI (blue). **H**: Infection of the RPE with Rcas-GFP control did not induce transdifferentiation. AMV3C2 staining represents the presence of the Rcas virus in the RPE. Dashed lines outline the RPE and areas of transdifferentiation. Note: For **C** and **D** only infected RPE responded by transdifferentiating. L represents lens; RPE represents retina pigmented epithelium; tr represents transdifferentiation; r represents retina. Infected mesenchyme (m) did not transdifferentiate. Scale bar in **H** represents 50 μm and applies to **A**-**H**.

It has previously been reported that activating the MEK/Erk pathway as well as ectopic expression of Pax6 can induce transdifferentiation of the chick RPE into neural retina during normal development of the RPE [10,23]. To show that MEK activation and ectopic Pax6 are able to induce transdifferentiation of the RPE after retinectomy, we injected either Rcas-MEK^DD^ (a constitutively active form of MEK) or Rcas-Pax6 into E4 eyes after retinectomy. We observed that in the absence of FGF2, RPE infected with either Rcas-MEK^DD^ or Rcas-Pax6, as shown by immunoreactivity to the antiviral antibody AMV3C2, was induced to transdifferentiate after three or seven days post-retinectomy, respectively (Figure 1C,D). Transdifferentiation is marked by a depigmented and thickened neuroepithelium that is continuous with the RPE (Figure 1B). These results suggest that MEK and Pax6 are possible targets of FGF signaling that can induce transdifferentiation. In addition, we used the photoreceptor marker visinin to show that the transdifferentiated RPE did indeed give rise to retinal neural cell types (Figure 1F,G). In other words, the RPE induced by MEK and Pax6 could dedifferentiate and proliferate to form a neuroepithelium that eventually differentiates into retinal cells.

It has also been previously reported that in vivo and in vitro Mitf overexpression leads to a hyperpigmented retinal phenotype [21,24,25]. However, it has not been demonstrated that Mitf overexpression is able to inhibit FGF2-stimulated transdifferentiation of the RPE in the E4 chick after retinectomy. To determine if Mitf can inhibit FGF2 stimulated transdifferentiation, we injected Rcas-Mitf into E4 retinectomized eyes along with an FGF2 bead. Overexpression of Mitf was able to inhibit FGF2-stimulated transdifferentiation. Transdifferentiation was seen only in areas where the RPE was not infected with the virus (Figure 1E). Therefore, our in vivo results in the E4 chick eye after retinectomy were consistent with results seen in both in vitro and in developing eyes of chick and quail.

As a control, we injected Rcas-GFP into the eye after retina removal at E4. We observed that infected RPE did not transdifferentiate after three days (Figure 1H).

### Temporal expression of Pax6 and the RPE markers Mitf and MMP115 during RPE development in the embryonic chick

To study the relationship between Pax6 and the RPE markers Mitf and MMP115 during the process of transdifferentiation, we believed it was important to determine the developmental expression of these molecules at different stages. Thus, we collected chick eyes at E3.5, E4, and E5 and assayed them by immunohistochemistry for protein expression in the RPE.

**Figure 2:**
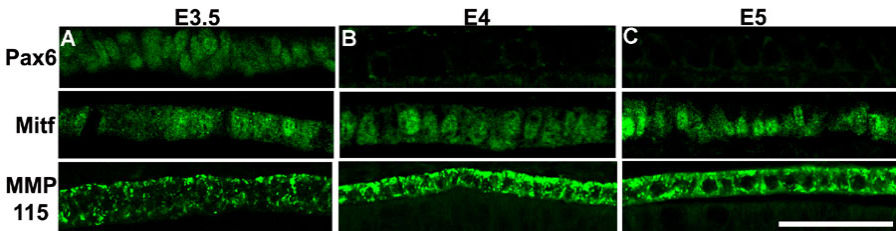
Expression of Pax6, Mitf, and MMP115 in the developing retinal pigment epithelium. **A**: Expression of Pax6 (top), Mitf (middle), and MMP115 (bottom) at E3.5. **B**: Expression of Pax6 (top), Mitf (middle), and MMP115 (bottom) at E4. **C**: Expression of Pax6 (top), Mitf (middle) and MMP115 (bottom) at E5. Scale bar represent 50 μm and applies to all panels.

At E3.5 the transcription factor, Pax6, is expressed in the RPE. Mitf, an RPE specific transcription factor, and MMP115, a marker for differentiated pigmented cells [26] are expressed in the RPE at E3.5 (Figure 2A). Figure 2 demonstrates that MMP115 is expressed at E3.5, but appears to be disorganized. By E4, Pax6 is almost gone from the RPE and by E5, Pax6 is not detectable in the RPE (Figure 2B,C). On the other hand, Mitf becomes more localized to the nucleus by E4 and is seen only in the nucleus at E5. MMP115 immunoreactivity becomes more intense and densely packed at both E4 and E5.

Thus, it appears that Pax6 is initially expressed during normal RPE development, but is downregulated as the RPE gets specified. At the same time, the RPE-specific proteins Mitf and MMP115 become more abundant and more organized (Figure 2).

### FGF2 causes a rapid increase in Pax6 expression

It is important to point out that for all figures used, pictures of the RPE were taken in areas where the RPE is close to the FGF2 or heparin (control) bead, except in the cases where no bead was added to the eye. Focusing on regions close to the bead eliminated results due to differences in FGF2 concentration that may arise from diffusion at different distances from the FGF2 source.

**Figure 3:**
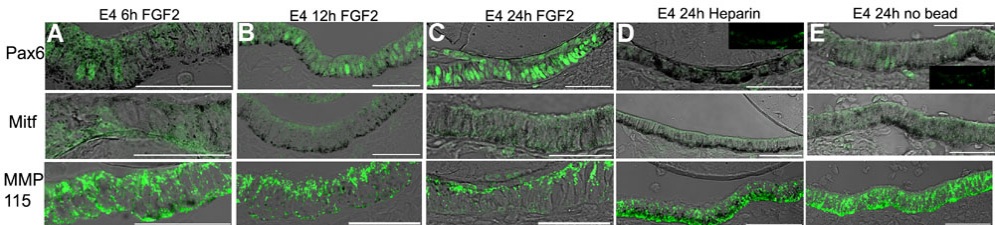
FGF2 stimulates Pax6 expression in the retinal pigment epithelium when the retina is removed at E4. **A**: Expression of Pax6 (top), Mitf (middle), and MMP115 (bottom) after retina removal at E4 and in response to 6 h of exposure to FGF2. **B**: Expression of Pax6 (top), Mitf (middle), and MMP115 (bottom) after retina removal at E4 and in response to 12 h of exposure to FGF2. **C**: Expression of Pax6 (top), Mitf (middle), and MMP115 (bottom) after retina removal at E4 and in response to 24 h of exposure to FGF2. **D**: Expression of Pax6 (top), Mitf (middle), and MMP115 (bottom) after retina removal at E4 and in response to 24 h of exposure to a heparin bead. Inset image shows Pax6 expression without DIC overlay. Note that few cells are Pax6 positive. **E**: Expression of Pax6 (top), Mitf (middle), and MMP115 (bottom) after retina removal at E4 with no bead and visualized 24 h post-retinectomy. Inset image shows Pax6 expression without DIC overlay. Note that Pax6 levels in no-bead and heparin bead controls are significantly lower than those treated with FGF2. Scale bars represent 50 μm. **A** was taken at a closer magnification than that of **B**-**E** (see scale bars).

We wanted to examine the relationship between the addition of FGF2 and Pax6, Mitf and MMP115 protein expression at early time points during the transdifferentiation process. To accomplish this, we performed retinectomies at approximately E4 and FGF2 beads or untreated heparin beads were placed into the optic cup. In addition, a no bead control was also included. Eyes were collected 4 (not shown), 6, 12, and 24 h after surgery and assayed by immunohistochemistry for Pax6, Mitf, and MMP115 expression. We observed that only when FGF2 was added to the optic cup after retinectomy did Pax6 protein levels start to increase in the RPE (Figure 3A). This is consistent with studies that have shown that Pax6 lies downstream of FGF signaling [23]. Six h after exposure to FGF2, there was a noticeable increase in Pax6 levels (Compare Figure 3A to control Figure 3D,E). As expected, only background staining was detected and no nuclear Mitf staining was observed in the RPE after 6 h. MMP115 was more disorganized than in a developing E4 RPE or in a control experiment (Compare Figure 2B to Figure 3A,D,E). Pax6 protein levels continued to increase in the RPE exposed to FGF2 in all other time points examined. At 12 and 24 h, Mitf was not detected in the RPE. In these cases, MMP115 immunoreactivity continued to decrease as the process of transdifferentiation persisted (Figure 3B,C).

As we expected, there were few Pax6-positive cells in the RPE 24 h after the retina had been removed and a heparin bead or no bead was placed in the optic cup (Figure 3D,E). Surprisingly, we observed in both cases that after 24 h, Mitf was not present in the RPE. This result was also consistent at different times (4 and 48 h, not shown). MMP115 protein was present in the RPE and was significantly more organized than the RPE of experimental eyes, where FGF2 was added for 24 h (Compare Figure 3D,E,C). Furthermore, we determined that there was no difference between control eyes with heparin beads or no beads. Therefore, for the rest of our experiments, we added heparin beads as a control.

### FGF2-stimulated Pax6 upregulation works through the MEK/Erk pathway and coincides with increased proliferation in the RPE during transdifferentiation

**Figure 4:**
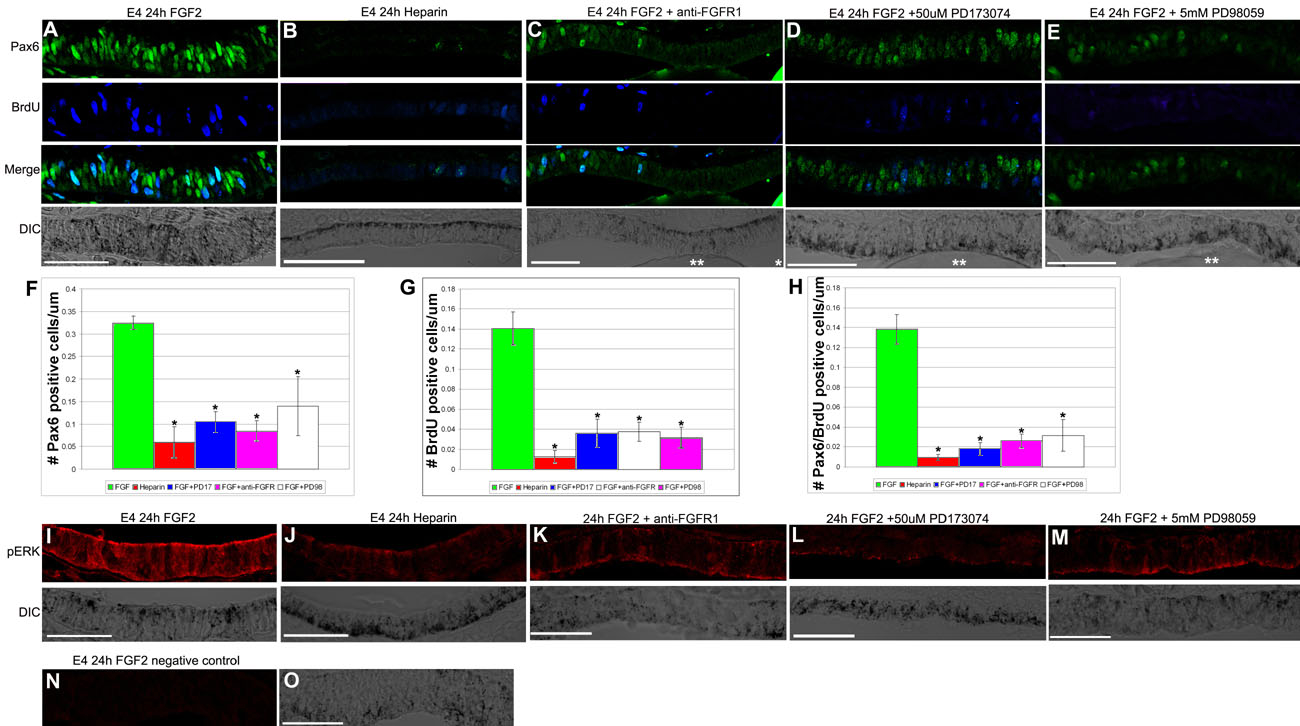
Pax6 and BrdU incorporation increase in response to FGF2 after retina removal at E4. **A**: Expression of Pax6 (top), BrdU (second), Pax6 and BrdU, Merge (third), and the DIC image (bottom) of the RPE after retina removal at E4 and 24 h of exposure to FGF2. **B**: Expression of Pax6 (top), BrdU (second), Pax6 and BrdU Merge (third) and the DIC image (bottom) of the RPE after retina removal at E4 and after 24 h of exposure to a heparin control bead. **C**: Expression of Pax6 (top), BrdU (second), Pax6 and BrdU Merge (third), and the DIC image (bottom) of the RPE after retina removal at E4 and after 24 h of exposure to FGF2 and FGFR1/FGFR2 blocking antibodies. **D**: Expression of Pax6 (top), BrdU (second), Pax6 and BrdU Merge (third), and the DIC image (bottom) of the RPE after retina removal at E4 and after 24 h of exposure to FGF2 and the FGFR inhibitor PD173074. **E**: Expression of Pax6 (top), BrdU (second), Pax6 and BrdU Merge (third) and the DIC image (bottom) of the RPE after retina removal at E4 and after 24 h of exposure to FGF2 and the MEK inhibitor PD98059. **F**: The number of Pax6-positive cells per μm of RPE in treatments shown in **A**-**E** was quantitated. The number of Pax6 positive cells was significantly reduced in all treatments compared to FGF alone. *p<0.01. **G**: The number of BrdU-positive cells per μm of RPE in treatments shown in **A**-**E** was quantitated. The number of BrdU positive cells was significantly reduced in all treatments compared to FGF alone. *p<0.01. **H**: The number of Pax6/BrdU positive cells per um of RPE in treatments shown in **A**-**E** was quantitated. The number of Pax6/BrdU positive cells was significantly reduced in all treatments compared to FGF alone. *p<0.01. **I**: Erk phosphorylation in the RPE after retina removal at E4 and after 24 h of exposure to FGF2 (top) and DIC image of the RPE (bottom). **J**: Erk phosphorylation in the RPE after retina removal at E4 and after 24 h of exposure to heparin (top) and DIC image of the RPE (bottom). **K**: Erk phosphorylation in the RPE after retina removal at E4 and after 24 h of exposure to FGF2 plus FGFR1/FGFR2-blocking antibodies (top) and DIC image of the RPE (bottom). **L**: Erk phosphorylation in the RPE after retina removal at E4 and after 24 h of exposure to FGF2 and the FGFR inhibitor PD173074 (top) and DIC image of the RPE (bottom). **M**: Erk phosphorylation in the RPE after retina removal at E4 and after 24 h of exposure to FGF2 plus the MEK inhibitor PD98059 (top) and DIC image of the RPE (bottom). **N**: Negative control for the immunostaining in panels **I**-**M**. **O**: DIC image of **N**. Scale bars represent 50 μm; ** represents FGF2 bead in **C**-**E**.

The process of transdifferentiation is marked by dedifferentiation and proliferation. To determine if the upregulation of Pax6 coincided with proliferation during the process of transdifferentiation, we performed retinectomies on E4 eyes and added FGF2 or control heparin beads. Additionally, 1 h before collecting the eyes, we added BrdU to the embryo to mark proliferating cells. Eyes were collected after 24 h and assayed for Pax6 expression as well as incorporation of BrdU. We found that similar to Pax6 protein levels, BrdU incorporation in the RPE was increased when compared to the RPE of control eyes (Compare Figure 4A,B). In the absence of FGF2, Pax6 protein was not detected and the cells in the RPE failed to proliferate (Figure 4B). This suggests that proliferation of the RPE is correlated with an upregulation of Pax6 only when ectopic FGF2 is added to the optic cup.

Our previous results have suggested that FGF2 signals through a MEK/Erk pathway [1], however, we have not conclusively demonstrated this. To test how FGF2 signals to stimulate transdifferentiation, we took advantage of the fact that FGF2 is able to upregulate Pax6 and BrdU incorporation after 24 h. To first show that FGF2 is indeed signaling through FGF receptors in order to stimulate transdifferentiation, we performed retinectomies at E4 and added an FGF2 bead along with an antibody that blocks the binding site of FGFR1 and FGFR2, or we added an inhibitor for all FGFRs, PD173074 (Figure 4C,D). As we expected, when we inhibited the FGFRs, Pax6 upregulation and BrdU incorporation seen after 24 h was significantly reduced (Figure 4C,D,F-H).

To show that the signaling events downstream of the FGF receptors include MEK/Erk activation, we used a potent and selective inhibitor of MEK, PD98059. We performed retinectomies on E4 eyes and added FGF2 as well as PD98059 and assayed for Pax6 and BrdU incorporation after 24 h. Similar to when we inhibited FGFRs, we found that PD98059 significantly inhibited Pax6 upregulation and BrdU incorporation associated with FGF2 stimulated transdifferentiation (Figure 4E,F-H).

To expand on our results, we also assayed for the active form of Erk, pErk, in the RPE after treatment with FGF2, heparin, FGF2 plus FGFR blocking antibodies, FGF2 plus PD173074, and FGF2 plus PD98059 (Figure 4I-M, respectively). Similar to our results with Pax6 and BrdU, we observed that FGF2 caused robust immunoreactivity of pErk in the RPE 24 h after removing the retina (Figure 4I). pErk immunoreactivity was barely detectable in heparin controls, suggesting that FGF2 leads to Erk phosphorylation (Figure 4J). We also observed that blocking FGF receptors or the downstream effector MEK was able to reduce FGF stimulated pErk immunoreactivity to control levels in the RPE (Figure 4K-M).

Combined, our results demonstrate that FGF2-stimulated transdifferentiation occurs through an FGF2/FGFR/MEK/Erk signaling cascade and leads to increased levels of pErk, an upregulation of Pax6 protein, and an increase in BrdU incorporation in the RPE.

### Rcas-Mitf does not interfere with FGF signaling to inhibit transdifferentiation

**Figure 5:**
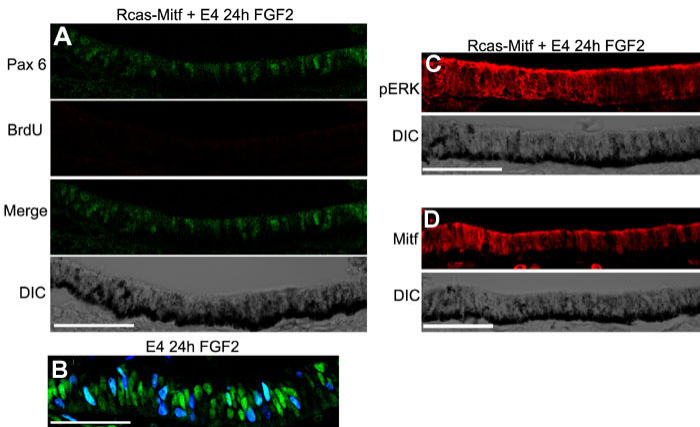
Mitf inhibits transdifferentiation by inhibiting Pax6, but not FGF signaling. **A**: Expression of Pax6 (top), BrdU (second), Pax6 and BrdU merge (third), and the DIC image (bottom) of the RPE after subretinally injecting Rcas-Mitf at E3, removing the retina at E4 and exposing the RPE to FGF2 for 24 h. **B**: Expression of Pax6 and BrdU in the RPE after 24 h of exposure to FGF2 and retina removal at E4. **C**: Erk phosphorylation in the RPE after subretinally injecting Rcas-Mitf at E3, removing the retina at E4 and exposing the RPE to FGF2 for 24 h (top), and the corresponding DIC image of the RPE (bottom). **D**: Mitf protein levels in the RPE after subretinally injecting Rcas-Mitf at E3, removing the retina at E4 and exposing the RPE to FGF2 for 24 h (top), and the corresponding DIC image of the RPE (bottom).

Our observation that Mitf protein was downregulated quickly after removal of the retina when no FGF2 was placed into the eye was surprising. Because of the data in published literature, we had expected to see Mitf protein present in the RPE until Pax6 levels increased (see discussion). However, we have also shown that Mitf is able to inhibit transdifferentiation stimulated by FGF2 (Figure 1C). To further characterize the ability of Mitf to inhibit transdifferentiation, we subretinally injected E3 chick eyes with Rcas-Mitf to allow the virus to infect the RPE. At E4 we removed the retina and added FGF2. Twenty-four hours later we collected the eyes and assayed for Pax6/BrdU incorporation and pErk levels. We found that Rcas-Mitf was able to inhibit FGF2-stimulated Pax6 upregulation and BrdU incorporation (Figure 5A). However, Rcas-Mitf did not inhibit the FGF2 stimulated increase in pErk (Figure 5B), suggesting that FGF signaling is occurring normally and Mitf is not inhibiting transdifferentiation by interfering with FGF signaling, but by most likely inhibiting Pax6. We also observed that in the Rcas-Mitf infected eyes, there were readily detectable levels of the Mitf protein after removing the retina and exposing the RPE to FGF2 for 24 h (Figure 5C). This is in stark contrast to uninfected eyes where no Mitf is seen in the RPE after retina removal regardless of the treatment (Figure 3).

These results demonstrate that while Mitf is downregulated in the RPE after retinectomy at E4 (Figure 3), it is still important for protecting the RPE against transdifferentiation by inhibiting the FGF2-stimulated Pax6 increase and simultaneous increase in proliferation.

## Discussion

### Spontaneous Mitf downregulation does not induce RPE transdifferentiation at E4

It has been well documented that in quail and mouse Mitf mutants, the RPE fails to develop properly and that the presumptive RPE instead develops into neural retina [8,18,20]. Nguyen and Arnheiter [8] demonstrated that as the eye is developing, the optic vesicle initially expresses both RPE and retina-specific genes, but signals from the surface ectoderm repress the RPE-specific gene Mitf such that the retina and RPE specific genes are expressed in distinct domains. They further demonstrated that the signals responsible for downregulating Mitf in the presumptive retina are likely FGF molecules being expressed by the surface ectoderm.

It has also been shown in the developing chick eye that when ectopic FGF8 is placed near the RPE at approximately E2, it is able to repress Mitf expression and cause transdifferentiation of the RPE into retina [9]. In mouse embryos, ectopic FGF9 expression is also able to induce RPE transdifferentiation, whereas embryos lacking FGF9 have RPE that extend into the presumptive neural retina [7].

Taking together the published data on the downregulation of Mitf by FGFs during eye development, we expected to observe dowregulation of Mitf in the RPE of chick eyes that underwent retinectomy only upon FGF addition, but not in its absence. We speculate that after retina removal, signals responsible for maintaining Mitf protein as well as transcript are disrupted. This could be due to stress signals produced after retina damage/removal or by disrupting cell-cell interactions between the retina and the RPE. In fact, the RPE is able to recover its Mitf protein expression 3 days post- retinectomy (not shown).

However, Fuhrmann et al. [27] have shown that Mitf and MMP115 expression in the embryonic chick eye are induced and maintained in the RPE by signals received from the extraocular mesenchyme. Similar to the Mitf mutant studies, Fuhrmann et al. [27] looked at early developmental events required for the initial induction and maintenance of Mitf in the developing RPE. Our studies, on the other hand, looked at E4 RPE that is more developed and differentiated and suggest that at later stages of RPE development, removing the retina is sufficient for Mitf downregulation, but that Mitf downregulation at this stage is not sufficient for induction of transdifferentiation. Recently, it has been shown that in E9 chick RPE cultures, Mitf-siRNA is effective at knocking down Mitf expression by 75% as well as reducing MMP115 levels by over 50%. At the same time these authors showed that Pax6 expression was increased [28]. However in this culture system, the RPE was only able to dedifferentiate, and not transdifferentiate. That is, the authors were not able to detect differentiation of the RPE into neurons. The authors found that downregulation of Mitf alone was not sufficient to induce transdifferentiation in vitro.

In our studies, we found that Mitf protein levels were low in the RPE after removing the retina, and that this reduction in Mitf levels was not correlated with increased Pax6 expression, BrdU incorporation, or transdifferentiation since we have previously demonstrated that retina removal alone does not yield any retina regeneration (Figure 1) [1]. It has been shown that in the silver quail Mitf mutant, the RPE is able to spontaneously transdifferentiate [18]. However, there are significant differences between studies performed by Mochii et al. [18] and ours. For example, those studies were performed on a developing quail eye, whereas our studies focus on a system for regeneration. Second, the spontaneous transdifferentiation observed by Mochii et al. [18] took place in the presence of the retina, which is able to provide growth factors. Coulombre and Coulombre [4] first demonstrated transdifferentiation of the RPE when they performed a retinectomy and placed a piece of retina back into the optic cup. It was later determined by Park and Hollenberg [5,6] that the factors produced by the retina that induced regeneration were likely FGFs. Indeed, several FGFs are produced in the developing chick retina [9,29,30]. So the spontaneous transdifferentiation observed in the quail mutants most likely had a combination of inactive Mitf and a source of FGFs to push the RPE to become neural retina. Another reason downregulation of Mitf may not lead to dedifferentiation or transdifferentiation is the presence of other RPE specific factors that are able maintain the RPE phenotype. For example, Otx2 has been shown to bind to the promoter region of some RPE specific genes controlled by Mitf and can work at the same hierarchical level to control RPE development [31].

Our observations that Mitf is downregulated after retina removal are intriguing, and the specific mechanism by which removing the retina leads to Mitf downregulation deserves attention in the future.

### FGF/FGFR/MEK/Erk signaling is required for transdifferentiation of the RPE at E4 in the embryonic chick

For the first time, we show that FGF2-induced transdifferentiation (in eyes undergoing retina regeneration) require FGF signaling through FGF receptors as well as the downstream effectors MEK and Erk. These signaling events ultimately lead to an increase in Pax6 protein, which is sufficient to induce transdifferentiation of the RPE. Results from Azuma et al. [23] corroborate our findings. This group demonstrated that in the absence of exogenous FGF2, ectopic Pax6 expression was sufficient to stimulate transdifferentiation. Additionally, it has recently been shown that Pax6 is required for induction of proliferation in the regenerating newt lens [32]. While our studies do not directly show that Pax6 is required for proliferation, they do show that Pax6 upregulation is sufficient for transdifferentiation, and in the absence of Pax6 upregulation, proliferation does not occur.

After retinectomy at E4, the increase in Pax6 does not need to overcome the Mitf protein in the RPE, since, as already discussed, it is spontaneously downregulated with retina removal. However, in the presence of ectopic Mitf expression using Rcas-Mitf, FGF-induced transdifferentiation is inhibited.

### Mitf inhibits transdifferentiation by interfering with Pax6 expression, not FGF signaling

A reciprocal relationship has been demonstrated between Mitf and Pax6. That is, when Mitf is inhibited, Pax6 levels in RPE increase and when Pax6 levels are ectopically increased, Mitf levels are decreased [21,28]. This reciprocal relationship may partly be due to the ability of Pax6 and Mitf to directly bind to one another and repress transactivational activity on their respective target promoters [22]. It has been previously shown that Mitf is able to inhibit Pax6 expression in chick RPE cells [21] and that Mitf overexpression in *Xenopus laevis* eyes results in induction of ectopic RPE [25].

Moreover, it has been shown that addition of FGF-coated beads near the optic vesicle leads to a rapid downregulation of Mitf in the developing mouse RPE at E9.5 [8]. Because of these previous studies demonstrating the relationship between Pax6 and Mitf, we were not surprised to find that Rcas-Mitf was able to inhibit Pax6 protein expression and BrdU incorporation after the RPE was exposed to FGF2 (Figure 5). However, we also show that the FGF signaling (measured by pErk) that induces Pax6 expression is not disrupted by this overexpression.

### Conclusion

In this study we show that Mitf is spontaneously downregulated in the RPE after retina removal at E4, and this decrease in Mitf protein is not sufficient for transdifferentiation to occur. Furthermore, ectopic FGF2 is required to drive Pax6 expression and induce transdifferentiation. The increased Pax6 expression is associated with increased BrdU incorporation. Finally, we show that the FGF/FGFR/MEK/Erk signaling cascade leads to an increase in Pax6 levels in the RPE, and that ectopic Mitf expression is sufficient to inhibit Pax6 expression but does not interfere with the activation of the FGF downstream effector Erk.

## References

[1] Spence JR, Madhavan M, Ewing JD, Jones DK, Lehman BM, Del Rio-Tsonis K (2004). The hedgehog pathway is a modulator of retina regeneration.. Development.

[2] Del Rio-Tsonis K, Tsonis PA (2003). Eye regeneration at the molecular age.. Dev Dyn.

[3] Tsonis PA, Del Rio-Tsonis K (2004). Lens and retina regeneration: transdifferentiation, stem cells and clinical applications.. Exp Eye Res.

[4] Coulombre JL, Coulombre AJ (1965). Regeneration of neural retina from the pigmented epithelium in the chick embryo.. Dev Biol.

[5] Park CM, Hollenberg MJ (1989). Basic fibroblast growth factor induces retinal regeneration in vivo.. Dev Biol.

[6] Park CM, Hollenberg MJ (1991). Induction of retinal regeneration in vivo by growth factors.. Dev Biol.

[7] Zhao S, Hung FC, Colvin JS, White A, Dai W, Lovicu FJ, Ornitz DM, Overbeek PA (2001). Patterning the optic neuroepithelium by FGF signaling and Ras activation.. Development.

[8] Nguyen M, Arnheiter H (2000). Signaling and transcriptional regulation in early mammalian eye development: a link between FGF and MITF.. Development.

[9] Vogel-Hopker A, Momose T, Rohrer H, Yasuda K, Ishihara L, Rapaport DH (2000). Multiple functions of fibroblast growth factor-8 (FGF-8) in chick eye development.. Mech Dev.

[10] Galy A, Neron B, Planque N, Saule S, Eychene A (2002). Activated MAPK/Erk kinase (MEK-1) induces transdifferentiation of pigmented epithelium into neural retina.. Dev Biol.

[11] Hill RE Favor J Hogan BL Ton CC Saunders GF Hanson IM Prosser J Jordan T Hastie ND van Heyningen V Mouse small eye results from mutations in a paired-like homeobox-containing gene. Nature 1991 354 522 5Erratum inNature 1992 355 750 1684639 10.1038/354522a0

[12] Burmeister M, Novak J, Liang MY, Basu S, Ploder L, Hawes NL, Vidgen D, Hoover F, Goldman D, Kalnins VI, Roderick TH, Taylor BA, Hankin MH, McInnes RR (1996). Ocular retardation mouse caused by Chx10 homeobox null allele: impaired retinal progenitor proliferation and bipolar cell differentiation.. Nat Genet.

[13] Barabino SM, Spada F, Cotelli F, Boncinelli E (1997). Inactivation of the zebrafish homologue of Chx10 by antisense oligonucleotides causes eye malformations similar to the ocular retardation phenotype.. Mech Dev.

[14] Ferda Percin E, Ploder LA, Yu JJ, Arici K, Horsford DJ, Rutherford A, Bapat B, Cox DW, Duncan AM, Kalnins VI, Kocak-Altintas A, Sowden JC, Traboulsi E, Sarfarazi M, McInnes RR (2000). Human microphthalmia associated with mutations in the retinal homeobox gene CHX10.. Nat Genet.

[15] Ashery-Padan R, Gruss P (2001). Pax6 lights-up the way for eye development.. Curr Opin Cell Biol.

[16] Marquardt T, Ashery-Padan R, Andrejewski N, Scardigli R, Guillemot F, Gruss P (2001). Pax6 is required for the multipotent state of retinal progenitor cells.. Cell.

[17] Horsford DJ, Nguyen MT, Sellar GC, Kothary R, Arnheiter H, McInnes RR (2005). Chx10 repression of Mitf is required for the maintenance of mammalian neuroretinal identity.. Development.

[18] Mochii M, Ono T, Matsubara Y, Eguchi G (1998). Spontaneous transdifferentiation of quail pigmented epithelial cell is accompanied by a mutation in the Mitf gene.. Dev Biol.

[19] Scholtz CL, Chan KK (1987). Complicated colobomatous microphthalmia in the microphthalmic (mi/mi) mouse.. Development.

[20] Bumsted KM, Barnstable CJ (2000). Dorsal retinal pigment epithelium differentiates as neural retina in the microphthalmia (mi/mi) mouse.. Invest Ophthalmol Vis Sci.

[21] Mochii M, Mazaki Y, Mizuno N, Hayashi H, Eguchi G (1998). Role of Mitf in differentiation and transdifferentiation of chicken pigmented epithelial cell.. Dev Biol.

[22] Planque N, Leconte L, Coquelle FM, Martin P, Saule S (2001). Specific Pax-6/microphthalmia transcription factor interactions involve their DNA-binding domains and inhibit transcriptional properties of both proteins.. J Biol Chem.

[23] Azuma N, Tadokoro K, Asaka A, Yamada M, Yamaguchi Y, Handa H, Matsushima S, Watanabe T, Kida Y, Ogura T, Torii M, Shimamura K, Nakafuku M (2005). Transdifferentiation of the retinal pigment epithelia to the neural retina by transfer of the Pax6 transcriptional factor.. Hum Mol Genet.

[24] Planque N, Raposo G, Leconte L, Anezo O, Martin P, Saule S (2004). Microphthalmia transcription factor induces both retinal pigmented epithelium and neural crest melanocytes from neuroretina cells.. J Biol Chem.

[25] Kumasaka M, Sato S, Yajima I, Goding CR, Yamamoto H (2005). Regulation of melanoblast and retinal pigment epithelium development by Xenopus laevis Mitf.. Dev Dyn.

[26] Mochii M, Agata K, Eguchi G (1991). Complete sequence and expression of a cDNA encoding a chicken 115-kDa melanosomal matrix protein.. Pigment Cell Res.

[27] Fuhrmann S, Levine EM, Reh TA (2000). Extraocular mesenchyme patterns the optic vesicle during early eye development in the embryonic chick.. Development.

[28] Iwakiri R, Kobayashi K, Okinami S, Kobayashi H (2005). Suppression of Mitf by small interfering RNA induces dedifferentiation of chick embryonic retinal pigment epithelium.. Exp Eye Res.

[29] Pittack C, Grunwald GB, Reh TA (1997). Fibroblast growth factors are necessary for neural retina but not pigmented epithelium differentiation in chick embryos.. Development.

[30] Desire L, Head MW, Fayein NA, Courtois Y, Jeanny JC (1998). Suppression of fibroblast growth factor 2 expression by antisense oligonucleotides inhibits embryonic chick neural retina cell differentiation and survival in vivo.. Dev Dyn.

[31] Martinez-Morales JR, Dolez V, Rodrigo I, Zaccarini R, Leconte L, Bovolenta P, Saule S (2003). OTX2 activates the molecular network underlying retina pigment epithelium differentiation.. J Biol Chem.

[32] Madhavan M, Haynes TL, Frisch NC, Call MK, Minich CM, Tsonis PA, Del Rio-Tsonis K (2006). The role of Pax-6 in lens regeneration.. Proc Natl Acad Sci USA.

